# Impact of Depression on Patients With Idiopathic Pulmonary Fibrosis

**DOI:** 10.3389/fmed.2020.00029

**Published:** 2020-02-07

**Authors:** Argyris Tzouvelekis, Theodoros Karampitsakos, Sofia Kourtidou, Evangelos Bouros, Vasilios Tzilas, Matthaios Katsaras, Chrysoula Antonou, Maria Dassiou, Demosthenes Bouros

**Affiliations:** First Academic Department of Pneumonology, Medical School, Hospital for Diseases of the Chest Sotiria, National and Kapodistrian University of Athens, Athens, Greece

**Keywords:** depression, idiopathic pulmonary fibrosis, prevalence, quality of life, antifibrotics

## Abstract

**Introduction:** Depression is prevalent in patients with Idiopathic Pulmonary Fibrosis (IPF). The impact of depression on quality of life and its correlation with disease severity in patients with IPF has not been thoroughly evaluated on prospective studies.

**Patients and Methods:** Between 2016 and 2017, we prospectively enrolled 101 patients (80 male, mean age (years) ± SD: 70.8 ± 8.1) with IPF (mean GAP score ± SD: 4.7 ± 1.8) without previous diagnosis of depression. Depressive symptoms were evaluated with Beck's depression inventory-II (BDI-II). Disease severity was evaluated with pulmonary function (FVC, DLCO) and exercise capacity measures. Symptom burden was assessed by cough and dyspnea scales. Health Related Quality of Life (HRQL) was assessed with two questionnaires.

**Results:** Data for analysis was available from 98 patients (97%). Forty two patients (42.9%) presented with depressive symptoms scoring≥14. A significant association between depressive symptoms and measures of: 1) disease severity: a) GAP score: *r* = 0.32, *p* = 0.007, b) DLCO: *r* = −0.28, *p* = 0.007, c) 6MWD: *r* = −0.39, *p* = 0.017, 2) symptom burden: a) cough: *r* = −0.57, *p* < 0.001, b) dyspnea (Borg: *r* = 0.54, *p* < 0.001, mMRC: *r* = 0.55, *p* < 0.001, SOBQ: *r* = 0.57, *p* < 0.001 and 3) HRQL: a) SGRQ: (Total score: *r* = 0.68, *p* < 0.001, Activity Score: *r* = 0.60, *p* < 0.001, Impact score: *r* = 0.68, *p* < 0.001, Symptoms score: *r* = 0.60, *p* < 0.001, b) K-BILD: *r* = −0.66, *p* < 0.001), was identified. There was no statistically significant difference in BDI-II (*p* = 0.62) and SGRQ (*p* = 0.64) 1 year after treatment with antifibrotics.

**Conclusions:** Patients with IPF and severe functional impairment tend to have increased risk for depression development and poor quality of life. Further prospective studies should investigate the role of antidepressant drug therapy in patients with IPF and comorbid depression.

## Introduction

Idiopathic Pulmonary Fibrosis (IPF) is a chronic, progressive fibrotic lung disease exerting a dramatic impact on patients' quality of life (1). The Food and Drug Administration approval of two novel anti-fibrotic compounds has finally shifted the therapeutic dial of IPF (2–5). While the emergence of drugs that slow down disease progression represents a major breakthrough in the management of IPF, pharmacological therapy does not address patient reported outcomes including cough, dyspnea and quality of life while at the same time comes with side-effects that have a net reduction in quality of life (1, 6). Importantly, IPF is an age-related disease and thus presents with major comorbidities that significantly impact survival and quality of life (7–9). Besides the traditionally recognized comorbidities of lung cancer, pulmonary hypertension and gastroesophageal reflux disease (7, 10–13), conditions related to the psychological impact of the disease have recently gained much of attention.

Anxiety occurs in approximately two-thirds of patients with IPF (14). Dyspnea, cough, sleep disturbance, loss of independence and feelings of social isolation coupled with the fear of forthcoming death due to the progressive and unpredictable course of the disease represent major causes of psychological distress eventually leading to depression. Epidemiologic studies demonstrated that prevalence of depression in patients with IPF ranged from 24.3 to 49.2% (15–19). Small retrospective studies have demonstrated a close association of depression with increased disease duration (9) and number of comorbidities (16). However, the association of depression with quality of life in patients with IPF has not been thoroughly evaluated on a prospective basis. More importantly, depressive symptoms following anti-fibrotic drugs initiation have not been studied yet. Pharmacological and cognitive-behavioral interventions could improve patients' quality of life (8, 20).

In this study, we aimed to prospectively evaluate the prevalence of depression in a single-center cohort comprised of 101 patients with IPF and we hypothesized that there might be an association of depressive symptoms with disease severity, symptom burden, health related quality of life (HRQL). Depressive symptoms in patients receiving disease-modifying antifibrotics have been also reported (21–26).

## Patients and Methods

Between 2016 and 2017, we prospectively enrolled 101 patients with IPF without previous diagnosis of depression. Diagnosis of IPF was based on 2011 ATS/ERS/JRS/ALAT guidelines (27). All patients provided written informed consent based on institutional guidelines. Prospective data collection and analysis was approved by the institutional review board of Hospital for Chest Diseases “Sotiria”, Athens, Greece (354/18-7-2017). Patients were enrolled during their first visit to our clinic between 2016 and 2017.

Depressive symptoms were evaluated with BDI-II. BDI-II was selected following a meticulous evaluation of all questionnaires from our center's psychologist (SK) and main researchers. The revised BDI-II is a questionnaire of 21 questions with answers being scored on a scale value of 0 to 3. Higher scores are indicative of more severe depression. Thus, patients are classified based on their answers into four groups: 0–13: minimal depression, 14–19: mild depression, 20–28: moderate depression, 29–63: severe depression. The revised BDI-II is a simple, fast, reliable questionnaire with psychometric and external validity (26). Furthermore, this 21-item-questionnaire corresponds to current DSM-IV Criteria for Major Depressive Disorder, while questions are of low risk for harming the participants psychologically or in any other dimension of health (28, 29). Recently, it has been applied as screening tool for depression and cognitive impairment in several chronic lung diseases including Chronic Obstructive Pulmonary Disease and IPF (25, 30).

IPF severity was evaluated with pulmonary function (Forced Vital Capacity: FVC, Diffusion capacity of lung for carbon monoxide: DLCO) and exercise capacity (6-minute walking test-MWT) measures, as well as with Gender-Age-Physiology (GAP) score. Symptom burden was assessed by dyspnea (Borg, modified Medical Research Council-mMRC, San Diego Shortness of Breath-SOBQ- questionnaire) and cough (Leicester cough questionnaire) scales. HRQL was assessed with Saint George's Research (SGRQ) and King's Brief Interstitial Lung Disease (K-BILD) questionnaires. All questionnaires were administered to the patients from our psychologist and two main researchers (AT, TK). Patients were kindly asked to fill questionnaires immediately in our clinic. Our psychologist and main researchers were available for possible queries from patients. Then, our psychologist performed a thorough discussion with patients in order to delineate their psychological status and suggest the appropriate management. Linear regression models were used to examine correlations between depression score and measures of disease severity, symptom burden and HRQL. In 30 patients, BDI-II and SGRQ was reassessed 1 year after antifibrotic treatment initiation.

## Results

### Baseline Characteristics

The baseline characteristics of patients enrolled in the study are summarized in Table 1. As demonstrated, data for analysis was available from 98 patients (97%). Eighty patients (82.5%) were male and 65 (64.3%) were ex-smokers. Mean FVC %pred ± SD and mean DLCO %pred ± SD were 77.0 ± 21.2 and 51.7 ± 19.4, respectively. Mean GAP score ± SD was 4.7 ± 1.8. None of the patients were taking antidepressants at the time of study (Datasheet 1).

**Table 1:** Baseline characteristics of patients included in study.

**Characteristics**	**Baseline data**
Total patients enrolled (*n*)	101
Patients data analyzed (*n*, %)	98 (97%)
Male/Female (*n*)	80/21
Age (years) (mean ± SD)	70.8 ± 8.1
Never smokers (*n*, %)	33 (32.6%)
Ex-smokers (*n*, %)	65 (64.3%)
Prior antidepressant treatment	0
FVC %pred (mean ± SD)	77.0 ± 21.2
DLco %pred (mean ± SD)	51.7 ± 19.4
6MWD (m) (mean ± SD)	401.8 ± 82.6
GAP score (mean ± SD)	4.7 ± 1.8
BDI-II score (mean ± SD)	13.7 ± 8.4
SGRQ score (mean ± SD)	39.7 ± 23.7
K-BILD (mean ± SD)	69.3 ± 18.7
BORG (mean ± SD)	3.4 ± 2.4
Leicester cough (mean ± SD)	107.6 ± 30.2
mMRC (mean ± SD)	1.9 ± 1.1

*BDI-II, Beck's Depression Inventory-II; DLco, Diffusion capacity of lung for carbon monoxide; FVC, Forced Vital Capacity; GAP, Gender-Age-Physiology; K-BILD, King's Brief Interstitial Lung Disease; mMRC, modified Medical Research Council; SD, Standard Deviation; SGRQ, Saint George's Research Questionnaire; 6MWD, 6-min walking distance*.

### Depression Prevalence and Severity

Mean BDI-II score ± SD was 13.7 ± 8.4. Forty two patients (42.9%) presented with depressive symptoms scoring≥14, indicating moderate-to-severe depression. In particular, classification of patients into four groups of depression severity based on their answers on BDI-II characterized 56 patients (57.1%) as minimally depressed (score 0–13). Twenty one patients (21.4%) were classified into mild depression group (score 14–19). Thirteen patients (13.3%) were moderately depressed (score 20–28). Finally, 8 patients (8.2%) presented with severe depression (score 29–63) (Table 2).

**Table 2:** Number of patients with IPF classified into each of four groups of depression severity based on their answers on BDI-II and their baseline characteristics.

**Depression severity**	**Patients** **(*N*, %)**	**Age** **(mean ± SD)**	**FVC%pred** **(mean ± SD)**	**DLco%pred** **(mean ± SD)**
Minimal depression (score 0–13)	56 (57.1%)	70.3 ± 8.3	81.3 ± 19.9	55.3 ± 19.0
Mild depression (score 14–19)	21 (21.4%)	70.9 ± 8.1	67.4 ± 19.1	48.0 ± 20.0
Moderate depression (score 20–28)	13 (13.3%)	72.8 ± 8.7	74.9 ± 24.0	41.3 ± 15.5
Severe depression (score 29–63)	8 (8.2%)	72.3 ± 7.9	64.0 ± 10.1	44.3 ± 22.2

### Significant Association of Depression With IPF Functional Severity, Symptom Burden, and HRQL

**Figure 1:**
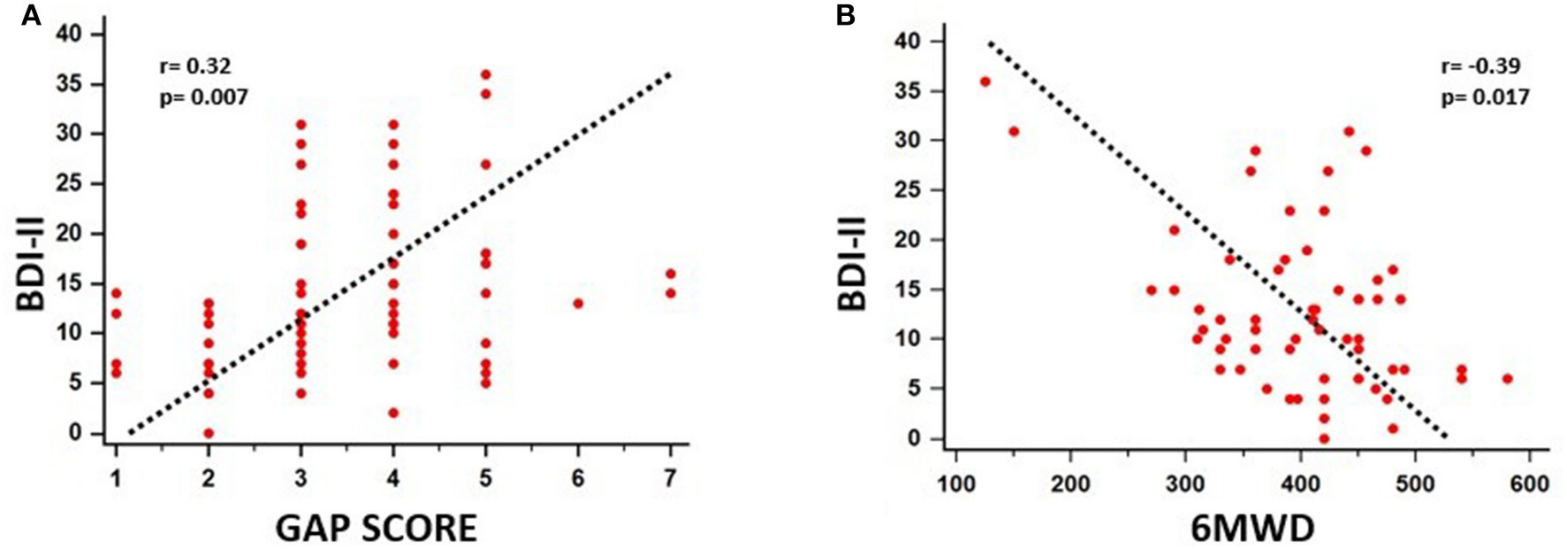
Significant association of depression with functional severity and exercise capacity impairment as assessed by GAP score (*r* = 0.32, *p* = 0.007) **(A)** and 6MWD (*r* = −0.39, *p* = 0.017) **(B)**, respectively.

**Figure 2:**
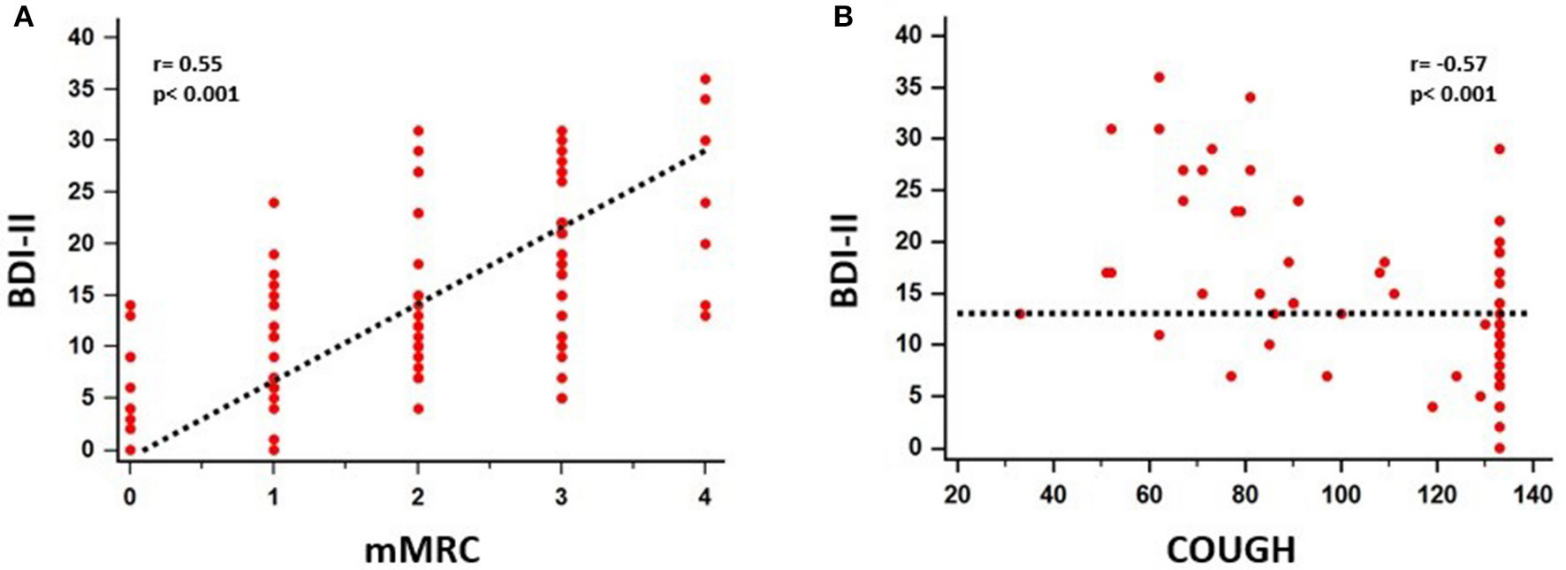
Significant association between depressive symptoms and mMRC (*r* = 0.55, *p* < 0.001) **(A)**, cough (*r* = −0.57, *p* < 0.001) **(B)** was identified.

**Figure 3:**
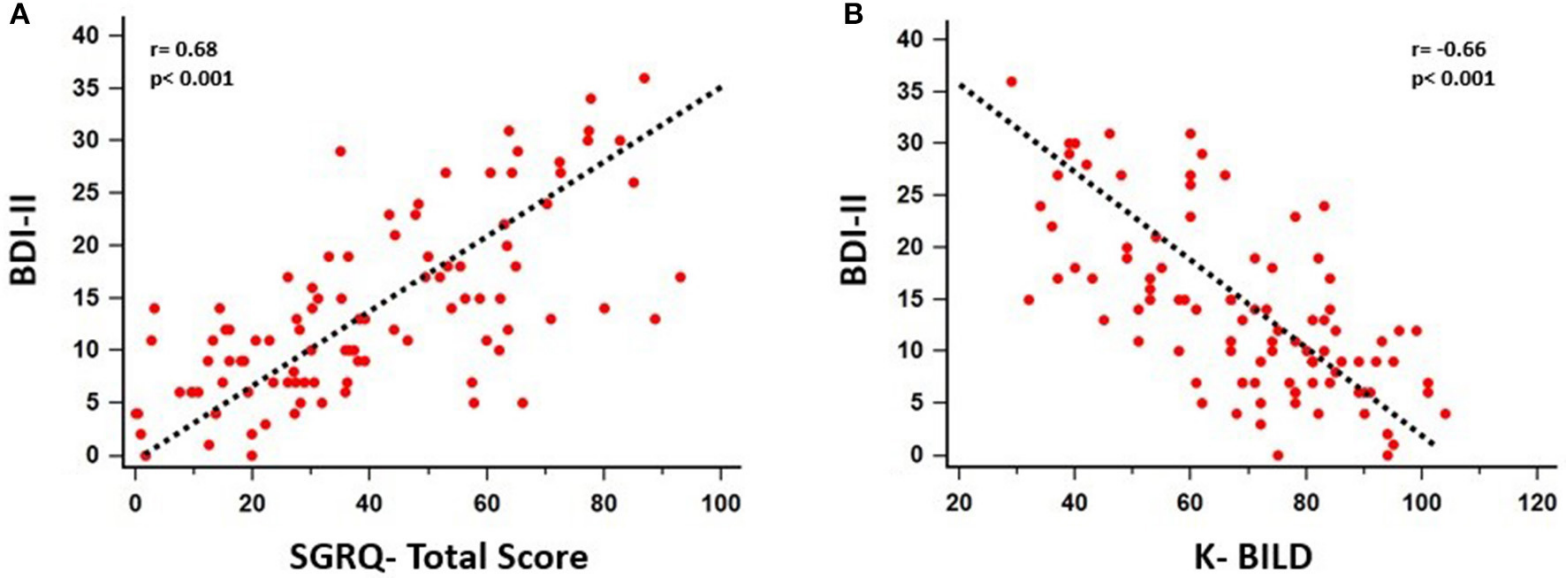
Significant association of depression with patients' quality of life as assessed by SGRQ (Total score: *r* = 0.68, *p* < 0.001, Activity Score: *r* = 0.60, *p* < 0.001, Impact score: *r* = 0.68, *p* < 0.001, Symptoms score: *r* = 0.60, *p* < 0.001) **(A)** and K-BILD (*r* = −0.66, *p* < 0.001) **(B)**.

A significant association between depressive symptoms and measures of functional severity, such as GAP score (*r* = 0.32, *p* = 0.007) and DLCO (*r* = −0.28, *p* = 0.007), was shown. Furthermore, depression severity was correlated with status of exercise capacity as assessed by 6MWD (*r* = −0.39, *p* = 0.017). Figure 1 schematically presents the significant association of depression with GAP score and 6MWD. Data for DLCO are not shown in figures. Depression severity had also a significant association with several indices of dyspnea, including Borg scale (*r* = 0.54, *p* < 0.001), and mMRC (*r* = 0.55, *p* < 0.001), SOBQ (*r* = 0.57, *p* < 0.001), as well as with cough, as indicated with Leicester questionnaire (*r* = −0.57, *p* < 0.001). Figure 2 schematically presents the association of depression with mMRC and cough. Data for Borg scale and SOBQ are shown in text and are not schematically presented. Depression symptoms showed a significant correlation with indicators of quality of life including: A) total score of SGRQ (*r* = 0.68, *p* < 0.001) as well as its counterparts (Activity score: *r* = 0.60, *p* < 0.001, Impact score: *r* = 0.68, *p* < 0.001, Symptoms score: *r* = 0.60, *p* < 0.001), B) K-BILD score (*r* = −0.66, *p* < 0.001). Figure 3 shows the significant association of depression with SGRQ and K-BILD. Finally, there was no association between severity of depression and age (*r* = 0.11, *p* = 0.3), smoking status (*r* = 0.05, *p* = 0.67) and gender (*r* = 0.19, *p* = 0.12) (data not shown in figures).

### Effect of Antifibrotic Treatment on BDI-II and HRQL

In 30 patients, depressive symptoms and HRQL were reassessed following 1 year of anti-fibrotic treatment through BDI-II and SGRQ, respectively. Sixteen patients had received nintedanib and 14 patients pirfenidone. With regards to SGRQ, we compared the change of this score in comparison with the suggested cut-off for clinical importance (change>4) and then we sought to determine if this change was statistically significant. Change in BDI-II following 1 year of anti-fibrotic treatment (ΔBDI-II) was−0.24 and change in SGRQ following 1 year of anti-fibrotic treatment (ΔSGRQ) was + 3.38. There was no statistically significant difference in BDI-II (*p* = 0.62) and SGRQ (*p* = 0.64) following treatment with antifibrotics. The change of SGRQ following 1 year of antifibrotics (+3.38) was also less than 4, which has been suggested as the minimum clinically important difference (Table 3).

**Table 3:** Median BDI-II and SGRQ at baseline and 1-year after initiation of antifibrotics in 30 patients.

**Variable**	***n***	***p*-value**
Baseline median BDI-II score	14.00 (95% CI: 10.76 to 22.23)	*p* = 0.62
1-year median BDI-II score	13.50 (95% CI: 9.00 to 20.82)	
ΔBDI-II	−0.24	
Baseline median SGRQ score	34.99 (95% CI: 27.41 to 49.38)	*p* = 0.64
1-year median SGRQ score	39.12 (95% CI: 29.60 to 51.81)	
ΔSGRQ	+3.38	

## Discussion

Our prospective study demonstrated that a considerable proportion of patients with IPF (42.9%) presented with major depressive symptoms. Importantly, this study showed that depression was indirectly associated with disease severity, symptom burden and quality of life in patients with IPF. This was also the first study investigating the effect of antifibrotics in depressive symptoms based on BDI-II. Given the prevalence of depression on patients with IPF, these results highlight the need for holistic care approaches focusing on cognitive behavioral therapy, pulmonary rehabilitation and anti-depressant medications in patients with IPF on a prospective basis.

Currently there is significant lack of knowledge on the ideal screening tool to detect depression in patients with IPF. We used BDI-II for the evaluation of depressive symptoms. Based on BDI-II, 42.9% of patients with IPF presented with depressive symptoms. In particular, 21.4, 13.3, and 8.2% of patients presented with mild, moderate and severe depression, respectively. These results are in line with existing literature recording prevalence of depression between 24.3 and 49.2% in patients with IPF (15–19). Despite epidemiologic association, there is currently a significant lack of knowledge supporting a direct pathogenetic association between lung fibrosis and depression; yet, it seems plausible that dyspnea, cough, referral for lung transplantation, reading of articles for median survival, sleep disturbance, loss of independence and feelings of social isolation could be among the reasons for depression development in patients with IPF (8).

After identifying depression as common in our IPF patient cohort, we sought to determine whether depression was associated with disease severity as indicated by physiological parameters including pulmonary functional tests and exercise capacity. Indeed, patients with higher scores in BDI-II presented with higher GAP score, lower DLCO and decline in 6MWD. A previous study assessing cognitive function in IPF recorded a direct association between severity of depression and advanced IPF, defined as DLCO<30% (30). Depression scores have been also correlated with baseline FVC%pred (18). A more recent study using Hospital Anxiety and Depression Scale (HADS) demonstrated no association between HADS and GAP score, in a small cohort of patients with IPF (15). Our findings for association of depression with disease severity could be explained by the fact that most of the aforementioned reasons for depression development become more obvious as disease progresses.

Following our observation that depression was almost linearly associated with physiological impairment in patients with IPF, we focused our analysis on patient-reported outcomes. Intriguingly, depression was positively associated with cough and dyspnea, as indicated by several questionnaires including Leicester as well as Borg scale, mMRC and SOBQ, respectively. Most studies are in line with our notion, except one recent report showing no association between depression and dyspnea grade or disease severity in IPF. This discrepancy might be due to the fact that baseline FVC%pred of the study was higher than all previous reports (15–17).

In line with previous studies (15, 31), we showed a significant association of depression with HRQL while no effect of anti-fibrotic treatment in patients' reported outcomes including depression was observed; yet, available data is underpowered and rigid conclusions cannot be drawn. On the other hand, our patients exhibited an increase in SGRQ following 1 year of anti-fibrotic treatment. This difference was near to the minimum clinically important difference of four; yet, it was not statistically significant. This change may be explained by both disease progression or potential drug-related adverse events (32) yet, our study was not designed for this purpose. With regards to SGRQ, we have to acknowledge that this questionnaire presents with major limitations particularly in the assessment of IPF-related quality of life as it was seminally designed for patients with obstructive lung diseases (33). Even though SGRQ holds acceptable validity and reliability in patients with IPF, some items (i.e., chest trouble and wheezing) are less relevant to the IPF patient group and possesses weaker psychometric properties. In addition, SGRQ has a complex scoring system that makes it difficult to use in routine clinical settings and its suboptimal internal consistency for the symptom domain may make it less sensitive to change. To overcome these limitations, an IPF-specific version of the SGRQ has been developed and showed significant associations with disease severity in a small cohort with patients with IPF; yet, larger prospective validation studies are sorely needed (34). With regards to changes in SGRQ following disease clinical course, the minimum clinically important differences (MCID) for SGRQ in patients with chronic lung diseases including COPD and IPF has been calculated around four (35). Nonetheless, these estimates are arbitrary and have been not been adequately validated to predict clinical outcomes. Baseline SGRQ scores greater than 30 have been associated with increased mortality (36); yet, other studies yielded contradictory results (37).

Importantly, our study was the first to investigate the impact of antifibrotics on disease-related psychological burden. Our finding that anti-fibrotic treatment has no effect on depression or quality of life is also in line with a prospective study showing no effect of nintedanib on dyspnea score (UCSD) and SGRQ (38). However, our analysis included a limited number of patients (*n* = 30) and thus findings should be interpreted with caution.

Our study exhibited a number of limitations that should be treated cautiously. Our patients were followed for a limited period of time and we did not report longitudinal outcomes. Another limitation was that BDI-II questionnaire is based on patients' answers and patients could hide their symptoms. We tried to overcome that caveat with the significant contribution of our center's psychologist. With regards to treatment modalities, our aim was to investigate the effect of antifibrotics. Data related to the effects of anti-fibrotics on depression was available for only a third of our patients; yet, our study was not designed to assess this issue on a prospective basis. Given that our aim was the investigation of antifibrotics effect, we have no data following antidepressant treatment. Finally, our results do not support the notion that depression has a direct impact on quality of life of patients with IPF, as our study lacks of validation cohort and a control group; yet, statistical associations of functional parameters of disease severity with patient reported outcomes related to depression and quality of life indicate that depression is prevalent in patients with IPF, may represent part of the vicious cycle of patient's deconditioning and thus may exert detrimental effects on life expectancy and quality of life by increasing the severity of dyspnoea or generating its own unique symptoms.

Our study adds further knowledge to the association of psychological burden with both clinical outcomes and quality of life of patients with IPF and highlight the urgent need for the involvement of palliative care in lung fibrosis. These studies are timely considering a global tendency to shift the patient toward the center of treatment and include patient-reported outcomes as primary objectives for clinical trials or as enrolment criteria for patients' screening. In line with this premise, one of the fewest positive studies, so far, enrolling patients with IPF was the administration of inhaled sodium cromoglicate for the treatment of chronic cough in patients with IPF (39). We and others, highlight the importance of applying holistic care approaches for the treatment of such complex and heterogeneous diseases, such as IPF (6). Assessment and management of comorbidities should be an integral part of our every-day clinical practice when dealing with patients with IPF, considering that almost 40% of these patients die from non-IPF causes (13). With regards to depression, it is now generally accepted that all patients, irrespective of disease severity should be carefully screened and monitored for depressive symptoms, during disease clinical course. Antidepressant regimens including cognitive behavioral therapy, pulmonary rehabilitation and anti-depressant medications in patients with IPF should be investigated in the context of large multi-center clinical trials (40–42). However, caution should be applied with the potential interaction of certain anti-depressants, such as fluvoxamine with pirfenidone (43). The findings that mirtazapine, a tetracyclic antidepressant agent, exerts anti-fibrotic properties in an experimental model of liver fibrosis (44) and serotonin is a pro-fibrotic mediator of experimental lung fibrosis (45) seem promising and intriguing.

In conclusion, we have shown that patients with IPF and severe functional impairment tend to have increased risk for development of severe depression and worse quality of life. Further prospective studies investigating the role of antidepressant regimens including cognitive behavioral therapy, pulmonary rehabilitation and anti-depressant medications in patients with IPF and comorbid depression are greatly anticipated.

## Data Availability Statement

The datasets generated for this study are available on request to the corresponding author.

## Ethics Statement

The studies involving human participants were reviewed and approved by Institutional review board of Hospital for Chest Diseases Sotiria, Athens, Greece. The patients/participants provided their written informed consent to participate in this study.

## Author Contributions

AT and TK performed the statistical analysis and wrote the manuscript. SK was the psychologist of our team. SK, EB, TK, AT, VT, MK, and MD were involved in data collection. DB supervised the study. All authors offered intellectual contribution for the manuscript and approved its final form.

### Conflict of Interest

The authors declare that the research was conducted in the absence of any commercial or financial relationships that could be construed as a potential conflict of interest.
