# Supplementary material for: Impact of Depression on Patients With Idiopathic Pulmonary Fibrosis
Source: Front Med (Lausanne). 2020 Feb 7;7:29. doi: 10.3389/fmed.2020.00029 (PMC7020231; doi:10.3389/fmed.2020.00029)
Supplement: Supplementary file 1 [file Data_Sheet_1.PDF]

Supplementary data

Impact of depression on patients with Idiopathic Pulmonary Fibrosis

Argyris Tzouvelekis[1*], Theodoros Karampitsakos[1*], Sofia Kourtidou[1], Evangelos Bouros[1], Vasilios Tzilas[1] , Matthaios Katsaras[1], Chrysoula Antonou[1], Maria Dassiou[1], Demosthenes Bouros[1]

[1] First Academic Department of Pneumonology, Hospital for Diseases of the Chest, "Sotiria", Medical School, National and Kapodistrian University of Athens, Athens, Greece

*these authors contributed equally to work

Correspondence:

Argyris Tzouvelekis

1st Academic Department of Respiratory Medicine,

National and Kapodistrian University of Athens,

Hospital for Diseases of the Chest, "Sotiria", Mesogion 152, 11527, Athens, Greece

argyrios.tzouvelekis@fleming.gr

BDI®-II

Date:

Name: ____________ Marital Status: ____________ Age: ________ Sex: __________
Occupation: ____________ Education: ____________

Instructions: This questionnaire consists of 21 groups of statements. Please read each group of statements carefully, and then pick the **one statement** in each group that best describes the way you have been feeling during the **past two weeks, including today**. Circle the number beside the statement you have picked. If several statements in the group seem to apply equally well, circle the highest number for that group. Make sure that you do not choose more than one statement for any group, including Item 16 (Changes in Sleeping Pattern) and Item 18 (Changes in Appetite).

1. Sadness

0 I do not feel sad.
1 I feel sad much of the time.
2 I am sad all the time.
3 I am so sad or unhappy that I can't stand it.

2. Pessimism

0 I am not discouraged about my future.
1 I feel more discouraged about my future than I used to be.
2 I do not expect things to work out for me.
3 I feel my future is hopeless and will only get worse.

3. Past Failure

0 I do not feel I am a failure.
1 I have failed more than I should have.
2 When I look back, I see a lot of failures.
3 I feel I am a total failure as a person.

4. Loss of Pleasure

0 I get as much pleasure as I ever did from the things I enjoy.
1 I don't enjoy things as much as I used to.
2 I get very little pleasure from the things I used to enjoy.
3 I can't get any pleasure from the things I used to enjoy.

5. Guilty Feelings

0 I don't feel particularly guilty.
1 I feel guilty over many things I have done or should have done.
2 I feel quite guilty most of the time.
3 I feel guilty all of the time.

6. Punishment Feelings

0 I don't feel I am being punished.
1 I feel I may be punished.
2 I expect to be punished.
3 I feel I am being punished.

7. Self-Dislike

0 I feel the same about myself as ever.
1 I have lost confidence in myself.
2 I am disappointed in myself.
3 I dislike myself.

8. Self-Criticalness

0 I don't criticize or blame myself more than usual.
1 I am more critical of myself than I used to be.
2 I criticize myself for all of my faults.
3 I blame myself for everything bad that happens.

9. Suicidal Thoughts or Wishes

0 I don't have any thoughts of killing myself.
1 I have thoughts of killing myself, but I would not carry them out.
2 I would like to kill myself.
3 I would kill myself if I had the chance.

10. Crying

0 I don't cry any more than I used to.
1 I cry more than I used to.
2 I cry over every little thing.
3 I feel like crying, but I can't.

***Beck Depression Inventory-II (BDI-II)*. Copyright © 1996 by Aaron T. Beck. Reproduced with permission of the publisher, NCS Pearson, Inc. All rights reserved.**

"*Beck Depression Inventory*" and "*BDI*" are trademarks in the US and/other countries, of Pearson Education, Inc. or its affiliate(s).

______ Subtotal Page 1 Continued on Back

11. Agitation

0 I am no more restless or wound up than usual.
1 I feel more restless or wound up than usual.
2 I am so restless or agitated that it's hard to stay still.
3 I am so restless or agitated that I have to keep moving or doing something.

12. Loss of Interest

0 I have not lost interest in other people or activities.
1 I am less interested in other people or things than before.
2 I have lost most of my interest in other people or things.
3 It's hard to get interested in anything.

13. Indecisiveness

0 I make decisions about as well as ever.
1 I find it more difficult to make decisions than usual.
2 I have much greater difficulty in making decisions than I used to.
3 I have trouble in making any decisions.

14. Worthlessness

0 I do not feel I am worthless.
1 I don't consider myself as worthwhile and useful as I used to.
2 I feel more worthless as compared to other people,.
3 I feel utterly worthless.

15. Loss of Energy

0 I have as much energy as ever.
1 I have less energy than I used to have.
2 I don't have enough energy to do very much.
3 I don't have enough energy to do anything.

16. Changes in Sleeping Pattern

0 I have not experienced any change in my sleeping pattern.

1a I sleep somewhat more than usual.
1b I sleep somewhat less than usual.

2a I sleep a lot more than usual.
2b I sleep a lot less than usual.

3a I sleep most of the day.
3b I wake up 1–2 hours early and can't get back to sleep.

17. Irritability

0 I am no more irritable than usual.
1 I am more irritable than usual.
2 I am much more irritable than usual.
3 I am irritable all the time.

18. Changes in Appetite

0 I have not experienced any change in my appetite.

1a My appetite is somewhat less than usual.
1b My appetite is somewhat greater than usual.

2a My appetite is much less than before.
2b My appetite is much greater than usual.

3a I have no appetite at all.
3b I crave food all the time.

19. Difficulty in Concentrating

0 I can concentrate as well as ever.
1 I can't concentrate as well as usual.
2 It's hard to keep my mind on anything for very long.
3 I find I can't concentrate on anything.

20. Tiredness or Fatigue

0 I am no more tired or fatigued than usual.
1 I get more tired or fatigued more easily than usual.
2 I am too tired or fatigued to do a lot of the things I used to do.
3 I am too tired or fatigued to do most of the things I used to do.

21. Loss of Interest in Sex

0 I have not noticed any recent change in my interest in sex.
1 I am less interested in sex than I used to be.
2 I am much less interested in sex now.
3 I have lost interest in sex completely.

***Beck Depression Inventory-II (BDI-II)*. Copyright © 1996 by Aaron T. Beck. Reproduced with permission of the publisher, NCS Pearson, Inc. All rights reserved.**

"*Beck Depression Inventory*" and "*BDI*" are trademarks in the US and/or other countries, of Pearson Education, Inc. or its affiliate(s).

______ Subtotal Page 2
______ Subtotal Page 1
______ Total Score

BDI®-II

Ημερομηνία:

Ονοματεπώνυμο: ______________________ Οικογενειακή κατάσταση: ____ Ηλικία: _____ Φύλο: ________
Επάγγελμα: ______________________ Εκπαίδευση: ______________________

Οδηγίες: Το ερωτηματολόγιο αυτό αποτελείται από 21 ενότητες. Διαβάστε προσεκτικά κάθε ενότητα προτάσεων και στη συνέχεια επιλέξτε από κάθε ομάδα **μία πρόταση**, η οποία περιγράφει καλύτερα τον τρόπο που αισθάνεστε κατά τη διάρκεια των **τελευταίων δύο εβδομάδων, συμπεριλαμβανομένης της σημερινής ημέρας.** Κυκλώστε τον αριθμό δίπλα από την πρόταση που επιλέξατε. Εάν πολλές προτάσεις κάποιας ενότητας σας φαίνονται ότι ισχύουν εξίσου, κυκλώστε το μεγαλύτερο αριθμό αυτής της ομάδας. Βεβαιωθείτε ότι δεν επιλέξατε περισσότερες από μία προτάσεις σε κάθε ενότητα, συμπεριλαμβανομένου του στοιχείου 16 (Αλλαγές στις Συνήθειες του Ύπνου) και του στοιχείου 18 (Αλλαγές στην Όρεξη).

1. Θλίψη

0 Δεν αισθάνομαι θλίψη.
1 Αισθάνομαι θλίψη τις περισσότερες φορές.
2 Είμαι συνεχώς θλιμμένος(-η).
3 Είμαι τόσο θλιμμένος(-η) ή δυστυχισμένος(-η) που δεν το αντέχω.

2. Απαισιοδοξία

0 Δεν είμαι απαισιόδοξος (-η) όσον αφορά το μέλλον μου.
1 Σε σχέση με το παρελθόν, αισθάνομαι πιο απαισιόδοξος(-η) για το μέλλον μου.
2 Δεν περιμένω τα πράγματα να πάνε καλά για εμένα.
3 Αισθάνομαι ότι δεν υπάρχει ελπίδα για το μέλλον μου και ότι όλα θα πάνε χειρότερα.

3. Αποτυχίες του παρελθόντος

0 Δεν αισθάνομαι αποτυχημένος(-η).
1 Απέτυχα περισσότερες φορές από όσες θα έπρεπε.
2 Όταν σκέφτομαιτο παρελθόν μου, βλέπω πολλές αποτυχίες.
3 Αισθάνομαι ότι ως άτομο είμαι εντελώς αποτυχημένο.

4. Απώλεια ευχαρίστησης

0 Αισθάνομαι την ίδια ευχαρίστηση που αισθανόμουν πάντα από τα πράγματα που μου αρέσουν.
1 Δεν απολαμβάνω αυτά που μου αρέσουν όσο τα απολάμβανα στο παρελθόν.
2 Αισθάνομαι ελάχιστη ευχαρίστηση από αυτά που μου άρεσαν στο παρελθόν.
3 Δεν αισθάνομαι καθόλου ευχαρίστηση από αυτά που μου άρεσαν στο παρελθόν.

5. Αισθήματα ενοχής

0 Δεν αισθάνομαι ιδιαίτερες ενοχές.
1 Αισθάνομαι ενοχές για πολλά πράγματα που έχω κάνει ή που θα έπρεπε να έχω κάνει.
2 Αισθάνομαι ενοχές τις περισσότερες φορές.
3 Αισθάνομαι πάντα ενοχές.

Beck Depression Inventory-II (BDI-II). Πνευματικά δικαιώματα © 1996 του Aaron T. Beck. Αναπαράγεται με την άδεια του εκδότη, NCS Pearson, Inc. Με επιφύλαξη κάθε νόμιμου δικαιώματος.

Οι ονομασίες "*Beck Depression Inventory*" και "*BDI*" αποτελούν εμπορικά σήματα της Pearson Education, Inc. ή των θυγατρικών της στις Ηνωμένες Πολιτείες ή/και σε άλλες χώρες.

______ Υποσύνολο σελίδας 1 Συνέχεια στην πίσω σελίδα

6. Αίσθημα τιμωρίας

0 Δεν αισθάνομαι ότι τιμωρούμαι.
1 Αισθάνομαι ότι ίσως να τιμωρούμαι.
2 Περιμένω ότι θα τιμωρηθώ.
3 Αισθάνομαι ότι τιμωρούμαι.

7. Δεν μου αρέσει ο εαυτός μου

0 Αισθάνομαι το ίδιο για τον εαυτό μου, όπως πάντα.
1 Έχω χάσει την εμπιστοσύνη στον εαυτό μου.
2 Είμαι απογοητευμένος(-η) από τον εαυτό μου.
3 Δεν μου αρέσει καθόλου ο εαυτός μου.

8. Αυτοκριτική

0 Δεν κατακρίνω ούτε κατηγορώ τον εαυτό μου πιο συχνά από ότι στο παρελθόν.
1 Κατακρίνω τον εαυτό μου περισσότερο από ότι στο παρελθόν.
2 Κατακρίνω τον εαυτό μου για όλα μου τα λάθη.
3 Κατηγορώ τον εαυτό μου για κάθε κακό που συμβαίνει.

9. Αυτοκτονικές σκέψεις ή επιθυμίες

0 Δε σκέφτομαι ποτέ την αυτοκτονία.
1 Σκέφτομαι την αυτοκτονία αλλά δεν πρόκειται να το πράξω .
2 Θα ήθελα να αυτοκτονήσω.
3 Θα αυτοκτονούσα εάν είχα την ευκαιρία.

10. Κλάμα

0 Δεν κλαίω περισσότερο από όσο έκλαιγα στο παρελθόν.
1 Κλαίω περισσότερο από όσο έκλαιγα στο παρελθόν.
2 Κλαίω με το παραμικρό.
3 Θέλω να κλάψω, αλλά δεν μπορώ.

11. Εκνευρισμός

0 Δεν εκνευρίζομαι ούτε ενοχλούμαι περισσότερο από ότι στο παρελθόν 1 Εκνευρίζομαι ή ενοχλούμαι περισσότερο από ότι στο παρελθόν.
2 Είμαι τόσο ανήσυχος(-η) ή εκνευρισμένος(-ή) που μου είναι αδύνατο να σταθώ ακίνητος(-η).
3 Είμαι τόσο ανήσυχος(-η) ή εκνευρισμένος(-ή) που πρέπει συνεχώς να κινούμαι ή να ασχολούμαι με κάτι.

12. Απώλεια ενδιαφέροντος

0 Δεν έχω χάσει τον ενδιαφέρον μου για άλλους ανθρώπους ή δραστηριότητες.
1 Ενδιαφέρομαι για άλλα πρόσωπα ή πράγματα λιγότερο σε σχέση με το παρελθόν.
2 Έχω χάσει σχεδόν κάθε ενδιαφέρον για άλλα πρόσωπα ή πράγματα.
3 Μου είναι δύσκολο να ενδιαφερθώ για οτιδήποτε.

13. Αναποφασιστικότητα

0 Λαμβάνω αποφάσεις το ίδιο καλά όπως πάντα.
1 Μου φαίνεται πιο δύσκολο, σε σχέση με το παρελθόν, να λαμβάνω αποφάσεις.
2 Έχω πολύ μεγαλύτερη δυσκολία, σε σχέση με το παρελθόν, να λαμβάνω αποφάσεις.
3 Δυσκολεύομαι να πάρω οποιαδήποτε απόφαση.

14. Αναξιότητα

0 Δεν αισθάνομαι ότι είμαι ανάξιος/α.
1 Δε θεωρώ ότι ο εαυτός μου αξίζει ή ότι είμαι τόσο χρήσιμος/η όσο ήμουν στο παρελθόν.
2 Αισθάνομαι ότι αξίζω λιγότερο από τους άλλους ανθρώπους.
3 Αισθάνομαι ότι δεν αξίζω τίποτα.

15. Απώλεια ενεργητικότητας

0 Έχω την ίδια ενεργητικότητα όπως πάντα.
1 Έχω λιγότερη ενεργητικότητα σε σχέση με το παρελθόν.
2 Δεν έχω αρκετή ενεργητικότητα για να κάνω πολλά πράγματα.
3 Δεν έχω καθόλου ενεργητικότητα για να κάνω οτιδήποτε.

16. Αλλαγές στις Συνήθειες Ύπνου

0 Δεν έχω διαπιστώσει καμία αλλαγή στις συνήθειες του ύπνου μου.
1a Κοιμάμαι κάπως περισσότερο από το συνηθισμένο.
1b Κοιμάμαι κάπως λιγότερο από το συνηθισμένο.
2a Κοιμάμαι πολύ περισσότερο από το συνηθισμένο.
2b Κοιμάμαι πολύ λιγότερο από το συνηθισμένο.
3a Κοιμάμαι κατά το μεγαλύτερο μέρος της ημέρας.

Beck Depression Inventory-II (BDI-II). **Πνευματικά δικαιώματα © 1996 του Aaron T. Beck. Αναπαράγεται με την άδεια του εκδότη, NCS Pearson, Inc. Με επιφύλαξη κάθε νόμιμου δικαιώματος.**

Οι ονομασίες “*Beck Depression Inventory*” και “*BDI*” αποτελούν εμπορικά σήματα της Pearson Education, Inc. ή των θυγατρικών της στις Ηνωμένες Πολιτείες ή/και σε άλλες χώρες.

______ Υποσύνολο σελίδας 2
______ Υποσύνολο σελίδας 1
______ Συνολική βαθμολογία

3b Ξυπνάω 1–2 ώρες νωρίτερα από ότι συνήθωςκαι μετά δε με ξαναπαίρνει ο ύπνος.

17. Ευερεθιστότητα

0 Δεν είμαι πιο ευερέθιστος(-η) από το συνηθισμένο.
1 Είμαι πιο ευερέθιστος(-η) από το συνηθισμένο.
2 Είμαι αρκετά πιο ευερέθιστος(-η) από το συνηθισμένο.
3 Είμαι συνεχώς ευερέθιστος(-η).

18. Αλλαγές στην όρεξη

0 Δεν διαπίστωσα καμία αλλαγή στην όρεξή μου.
1a Η όρεξή μου είναι κάπως μικρότερη από το συνηθισμένο.
1b Η όρεξή μου είναι κάπως μεγαλύτερη από το συνηθισμένο.
2a Η όρεξή μου είναι πολύ μικρότερη σε σύγκριση με το παρελθόν.
2b Η όρεξή μου είναι πολύ μεγαλύτερη από το συνηθισμένο.
3a Δεν έχω καθόλου όρεξη.
3b Θέλω συνέχεια κάτι να τρώω.

19. Δυσκολία συγκέντρωσης

0 Μπορώ να συγκεντρωθώ το ίδιο καλά όπως παλιά.
1 Δεν μπορώ να συγκεντρωθώ τόσο καλά όπως συνήθως.
2 Μου είναι δύσκολο να συγκεντρωθώ σε κάτι για μεγάλο χρονικό διάστημα.
3 Διαπιστώνω ότι δεν μπορώ να συγκεντρωθώ σε τίποτα.

20. Κούραση ή κόπωση

0 Δεν αισθάνομαι περισσότερη κόπωση ή κούραση σε σχέση με το συνηθισμένο.
1 Αισθάνομαι περισσότερη κόπωση ή κούραση πιο εύκολα σε σχέση με παλαιότερα.
2 Αισθάνομαι πολλή κόπωση ή κούραση για να κάνω πολλά πράγματα που συνήθιζα να κάνω στο παρελθόν.
3 Αισθάνομαι υπερβολική κόπωση ή κούραση για να κάνω τα περισσότερα από όσα έκανα στο παρελθόν.

21. Απώλεια σεξουαλικού ενδιαφέροντος

0 Δεν έχω διαπιστώσει καμία πρόσφατη αλλαγή στο ενδιαφέρον μου για το σεξ.
1 Με ενδιαφέρει λιγότερο το σεξ από ότι άλλοτε.
2 Με ενδιαφέρει πολύ λιγότερο το σεξ τώρα.
3 Έχω χάσει εντελώς κάθε ενδιαφέρον για το σεξ.

***Beck Depression Inventory-II (BDI-II)*. Πνευματικά δικαιώματα © 1996 του Aaron T. Beck. Αναπαράγεται με την άδεια του εκδότη, NCS Pearson, Inc. Με επιφύλαξη κάθε νόμιμου δικαιώματος.**

Οι ονομασίες “*Beck Depression Inventory*” και “*BDI*” αποτελούν εμπορικά σήματα της Pearson Education, Inc. ή των θυγατρικών της στις Ηνωμένες Πολιτείες ή/και σε άλλες χώρες.

______ Υποσύνολο σελίδας 3
______ Υποσύνολο σελίδας 1
______ Συνολική βαθμολογία
